# *Ex-Vivo* Dynamic 3-D Culture of Human Tissues in the RCCS™ Bioreactor Allows the Study of Multiple Myeloma Biology and Response to Therapy

**DOI:** 10.1371/journal.pone.0071613

**Published:** 2013-08-26

**Authors:** Marina Ferrarini, Nathalie Steimberg, Maurilio Ponzoni, Daniela Belloni, Angiola Berenzi, Stefania Girlanda, Federico Caligaris-Cappio, Giovanna Mazzoleni, Elisabetta Ferrero

**Affiliations:** 1 Department of Oncology, San Raffaele Scientific Institute, Milan, Italy; 2 Laboratory of Tissue Engineering, Department of Clinical and Experimental Sciences, School of Medicine, University of Brescia, Brescia, Italy; 3 Pathology Unit, San Raffaele Scientific Institute, Milan, Italy; 4 Institute of Pathological Anatomy, Department of Clinical and Experimental Sciences, School of Medicine, University of Brescia, Brescia, Italy; 5 Bone Marrow Transplantation Unit, San Raffaele Scientific Institute, Milan, Italy; 6 Vita-Salute San Raffaele University School of Medicine, Milan, Italy; Texas A&M University, United States of America

## Abstract

Three-dimensional (3-D) culture models are emerging as invaluable tools in tumor biology, since they reproduce tissue-specific structural features and cell-cell interactions more accurately than conventional 2-D cultures. Multiple Myeloma, which depends on myeloma cell-Bone Marrow microenvironment interactions for development and response to drugs, may particularly benefit from such an approach. An innovative 3-D dynamic culture model based on the use of the RCCS™ Bioreactor was developed to allow long-term culture of myeloma tissue explants. This model was first validated with normal and pathological explants, then applied to tissues from myeloma patients. In all cases, histological examination demonstrated maintenance of viable myeloma cells inside their native microenvironment, with an overall well preserved histo-architecture including bone lamellae and vessels. This system was then successfully applied to evaluate the cytotoxic effects exerted by the proteasome inhibitor Bortezomib not only on myeloma cells but also on angiogenic vessels. Moreover, as surrogate markers of specialized functions expressed by myeloma cells and microenvironment, β2 microglobulin, VEGF and Angiopoietin-2 levels, as well as Matrix Metalloproteases activity, were evaluated in supernatants from 3D cultures and their levels reflected the effects of Bortezomib treatment. Notably, determination of β2 microglobulin levels in supernatants from Bortezomib-treated samples and in patients'sera following Bortezomib-based therapies disclosed an overall concordance in the response to the drug *ex vivo* and *in vivo*.

Our findings indicate, as a proof of principle, that 3-D, RCCS™ bioreactor-based culture of tissue explants can be exploited for studying myeloma biology and for a pre-clinical approach to patient-targeted therapy.

## Introduction

Multiple Myeloma (MM) is a plasma cell (PC) tumor. Tight interactions between MM cells and Bone Marrow (BM) microenvironment are central to MM pathogenesis as they allow the delivery of pro-survival signals and confer chemo-resistance to neoplastic cells [1]–[3]. Indeed, tumor microenvironment is emerging as a major determinant in tumor initiation, progression and response to therapy in both haematological [4] and solid tumors [5], [6]. Hence, the generation of *in vitro* models able to recapitulate tumor microenvironment is critical for both understanding cancer biology and assessing its vulnerability to drugs [7]. While *in vivo* tissues and organs are three- dimensional (3-D), our capacity to understand their formation, function, and pathology traditionally relied on two-dimensional (2-D) cell culture models [8]. Indeed, recent studies documented differences between cell morphology and behaviour in static, 2D formats compared with more physiological 3D microenvironments [7], [9], [10]. Accordingly, 3-D approaches, which better reproduce *in vivo*-like responses, have become a focus of intense investigation [11]–[13]. In this context, a major breakthrough in the study of MM was achieved by Kirshner et al. [14], who proposed a new 3-D culture model, which enables clonal expansion of MM PC as well as testing of anti-tumor drugs.

Currently available 3-D systems include *ex vivo* models, based on the use of isolated cells and tissue-derived components (tissue engineering methods). However, these models do not fully reproduce the complexity and specificity of the native tissue architecture. This is especially true for the vascular compartment, that critically contributes to MM progression, thus representing a major target for anti-MM therapies [15]–[18]. As an example, the proteasome inhibitor Bortezomib is considered to be an effective anti-MM drug, not only for its cytotoxic effect against MM cells, but also for its anti-angiogenic activity [19]–[22]. Culture of tissue explants in static condition is an alternative 3-D system, but the reduced mass transport (gas/nutritional support and waste removal) achieved [23] limits its application to morpho-functional analyses.

To circumvent these limitations and to provide new models that more closely resemble the *in vivo* situation in patients, we applied a 3-D dynamic culture system, based on the use of the innovative microgravity technology provided by the Rotary Cell Culture System (RCCS™) Bioreactor, to *ex-vivo* culture of MM specimens. In this dynamic, horizontally rotating bioreactor, the culture vessel is fluid-filled, so that no headspace is left between atmosphere and culture medium. All during the experimental procedures, RCCS™ operational conditions were constantly monitored and optimized for obtaining a laminar flow of the fluid medium inside the culture chamber, while the 3D tissue samples were maintained suspended in a relatively stable position (condition of “free fall”), so that shear forces and turbulence normally associated with impeller-driven stirred bioreactors were reduced to a minimum. These hydrodynamic conditions related to the culture vessel rotation allow also to offset tissue sedimentation. Finally, the peculiar structure of the vessel (that includes a gas exchange membrane) favours the optimal oxygenation of thick, 3D samples, reducing the occurrence of the central core necrosis often observed with other bioreactors. The main advantages of our culture model, in comparison to conventional 2-D and 3-D static culture systems, are presented in the Figure S1.

By providing an optimal balance between increased mass transfer and deleterious effects of shear stress, this dynamic bioreactor, as already demonstrated by our group, generates the best conditions for long-term *in vitro* maintenance of cell viability and function in 3-D cell culture systems, multicellular engineered tissue-like constructs, and tissue explants [9], [24]. Thus, our strategy was to capitalize the advantages offered by the RCCS™ bioreactor to the culture of MM tissues, and particularly of human BM samples, whose complexity is not fully reproduced by currently available models.

The RCCS™ Bioreactor was firstly evaluated for its ability to preserve skin architecture and blood vessel integrity. The system was further validated for its capacity to maintain the complex bone/BM microenvironment with whole rat tibia epiphyses, and also bone explants from healthy donors. We finally applied the same strategy to tissue explants from MM patients, including BM and extra-medullary localization. Herein we demonstrate, as a proof of principle, that our dynamic, 3-D culture model allows to explore, for extended time periods, morphological and functional features of MM tissue components (vessels included) as well as their specific sensitivity to drug(s).

## Materials and Methods

### Patients

Tissue explants were obtained from five MM patients and included BM biopsies obtained at vertebroplasty for patient 1, 2 and 3, skull bone from patient 4, and a skin lesion from patient 5. BM biospsies from three elderly patients undergoing hip replacement (HD1, HD2, HD3) and a 14-year-old boy undergoing knee arthroplasty were used as controls. Skin samples were obtained from patients undergoing abdominal surgery. Renal cell carcinoma (RCC) samples were obtained from patients undergoing tumor resection. Written informed consent was obtained from patients (or from the parents in the case of the minor), in accordance with the Declaration of Helsinki and the approval for use of primary samples was obtained from the Institutional Review Board of the San Raffaele Scientific Institute.

Patient 1 was a forty-year-old man, newly diagnosed with MM. Patient 2 was a 66-year-old man with a 3-year history of micromolecular MM, who developed new osteolytic lesions requiring vertebroplasty. Patient 3 was a 75-year-old man with a not-secretory myeloma evolved from plasmocytoma. These three patients did not receive any therapy at the time when vertebroplasty was performed.

Patient 4 was a 61-year-old woman who developed a subcutaneous frontal mass infiltrating the right orbit. Histological examination of the lesion showed neoplastic PC monoclonal for λ light chain. She was treated with thalidomide plus dexamethasone and autologous hematopoietic stem cell transplantation (HSCT) and she died after two weeks of pneumonitis and septic shock. We obtained tissue biopsies from skull bone at the diagnosis, before any medical treatment.

Patient 5 was a 62-year-old man with a 3-year history of micromolecular MM. Despite different lines of therapy, including Bortezomib-based therapy, autologous HSCT and allogeneic HSCT from matched unrelated donor (MUD), the disease progressed and the patient developed subcutaneous abdominal localizations, which were biopsied for diagnosis and submitted to culture in Bioreactor. Histology showed infiltration with monoclonal PC expressing λ light chain. A salvage therapy with Bortezomib, liposomal doxorubicin and dexamethasone was started, but the patient developed invasive aspergillosis and Staphylococcus epidermidis sepsis and died nine months after allogeneic HSCT.

Baseline characteristics of MM patients are summarized in Table 1.

**Table 1:** Demographic and clinical characteristics of MM patients.

Patient	Age/sex	Diagnosis	DSS	ISS	Therapies before biopsy	Tissue
Pt1	40/M	MM IgGλ	IIIA	3	None	BM
Pt 2	66/M	MM µmolλ	IIA	3	Btz+Doxo; ARA-C, Btz+Mel+auto-HSCT	BM
Pt 3	75/M	MM nsλ	IIIA	3	Mel, Prednisone, Thal	BM
Pt 4	61/F	MM IgG λ	IIIA	3	None	Skull
Pt 5	62/M	MM µmol λ	IIIA	nk	Thal/Dex+double auto-HSCT; Btz/Dex+Mel+third auto-HSCT; Btz/Dex/Doxo+MUD transpl.	Skin

Abbreviations: Thal. thalidomide; Btz, Bortezomib; Dex, Dexamethasone; Doxo, Doxorubicin; Mel, Melphalan; nk, not known; ns, not secretory; µmol, micromolecular.

### Animals

Young (7–8 week­old) male Sprague­Dawley rats (Harlan­Europe, Milan, Italy) whose body weight ranged from 200 to 225 g were used as bone donors, in accordance with Italian National Guidelines for the care and use of laboratory animals. Tibial bones were collected, processed and cut as described [24]. Studies on animal models were approved by the Ethical Committee of the San Raffaele Scientific Institute and carried out according to the prescribed guidelines.

### Tissue Cultures in RCCS™ Bioreactor

3-D, dynamic culture of biopsies was performed using the RCCS™ bioreactor RCCS-1 (Synthecon Inc., Houston TX, USA), in 10 ml-HARV culture vessels, equipped with a gas exchange membrane made of silicon rubber, that allows an optimal diffusion of oxygen inside the vessel [24]. Bioreactor was kept inside an incubator, with humidified atmosphere at 37°C and 95% air 5% CO_2_. During the experimental procedures, RCCS™ operational conditions, settled in order to keep samples in a “free fall” condition that maximise mass transfer, were constantly monitored, in order to reduce to a minimum the mechanical stress (shear forces) on the explants surface and to prevent samples sedimentation. Normal skin and bone, as well as RCC and MM explants (2–3 mm^3^ of maximal volume) were cultured for three up to 14 days in RPMI plus 10% Fetal Calf Serum (tissue culture medium, TCM). To evaluate the effects of Bortezomib (PS-341, Velcade®, Millenium Pharmaceuticals, Cambridge MA) on MM tissue, the drug was added at 50 nM to parallel cultures. Tissue explants were harvested at 3–4 days intervals, formalin-fixed, decalcified and paraffin-embedded. Concomitantly, supernatants from culture chambers were withdrawn and frozen at −20°C until use, and fresh TCM was added. As a control, when indicated, parallel cultures of normal skin and bone explants as well as of RCC in static conditions were performed in Petri dishes and maintained under the same culture conditions as for cultures in Bioreactor.

### Histochemistry and Immunohistochemistry

Serial 5 µm thick sections were stained with hematoxylin and eosin (H&E) and with anti-CD34 mAb (QBEnd/10, Novocastra, Newcastle upon Tyne, UK), as previously described [25]. Anti-Podoplanin, Clone D2-40 (DAKO) was used to identify lymphatic vessels. Microvascular density (MVD) quantification was performed as described [26].

### Determination in supernatants of β2 microglobulin, VEGF, Angiopoietin-2, and of metalloproteases (MMP)-2 and -9

Supernatants from untreated or Bortezomib-treated MM explants and bone controls were serially retrieved and frozen. Samples were subjected to SDS-PAGE in 10% polyacrylamide gel, containing 1 mg/ml gelatine (Sigma), to assess MMP-2 and MMP-9 activities as described [27]. VEGF and Angiopoietin-2 (Ang-2) concentrations were determined by ELISA. β2 microglobulin concentration was determined by particle enhanced immunonephelometry.

### Cell isolation and apoptosis

PC were isolated from Patient 4's skull lesion by density centrifugation [28]. PC death upon 24-hrs *in vitro* treatment with Bortezomib 50 nM was assessed by propidium iodide (PI) staining and FACS analysis [29] and by Transmission Electron Microscopy (TEM), as described [30].

### Statistical analysis

Correlation between β2 microglobulin, VEGF and Ang-2 levels in supernatants from five MM patients (either treated or untreated with Bortezomib) was assessed using the Spearman's rho coefficient (*r*). A p-value of ≤0.05 was considered statistically significant.

## Results

### RCCS™ Bioreactor preserves tissue architecture and blood vessels integrity

**Figure 1:**
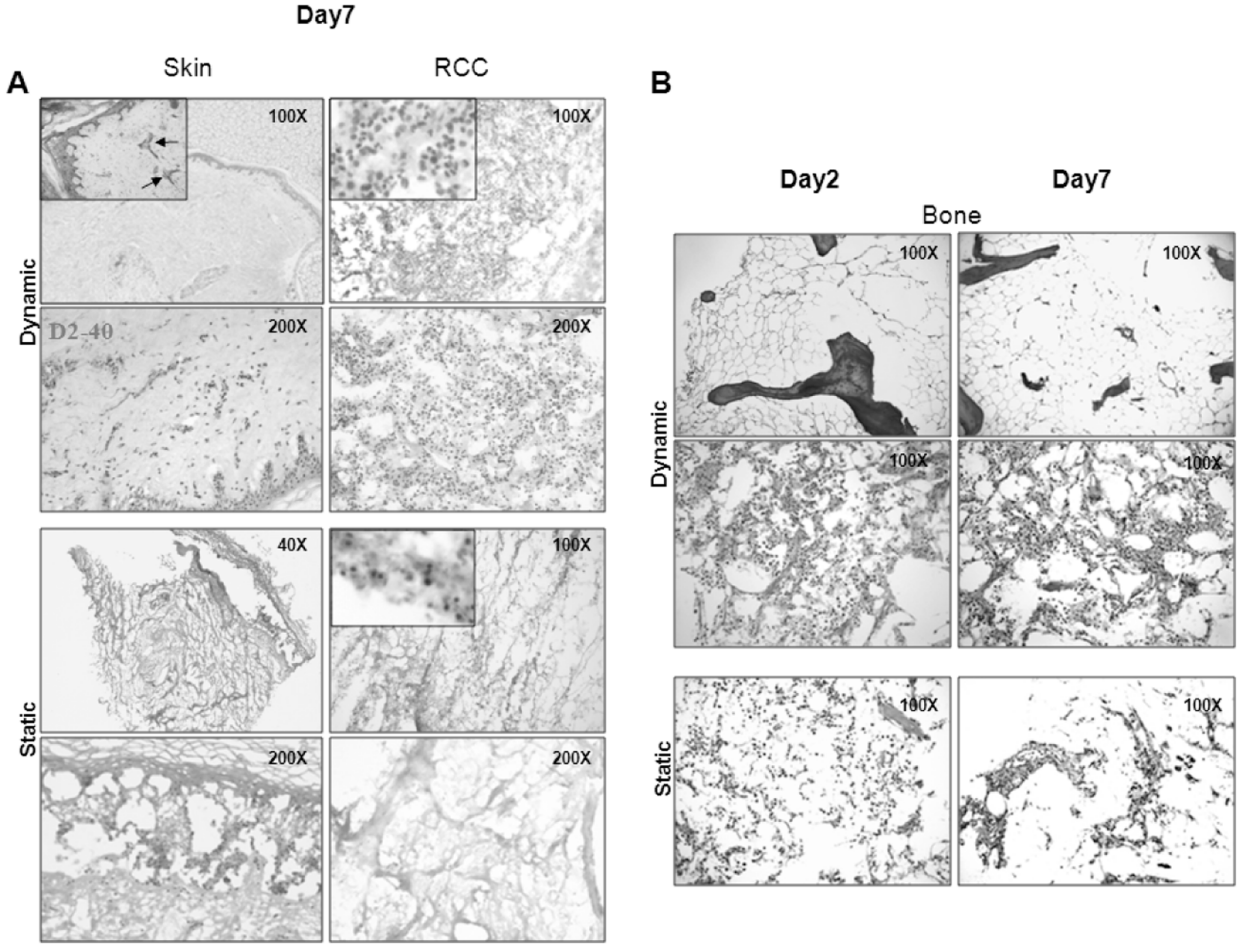
3-D culture in the RCCS™ bioreactor preserves cellularity and tissue histo-architecture. A. Left: skin explants from healthy donors were cultured in the RCCS™ bioreactor (upper panels) and in static conditions (lower panels) for seven days. Epidermal and dermal strata and annexes are well preserved and vessels, both blood (insert, arrows) and lymphatic (D2-40) are identifiable in dynamic conditions (upper panels), while histo-architecture is completely disrupted in static conditions (lower panels). Right: RCC samples cultured for seven days in Bioreactor (upper panels) showed maintenance of cellularity and viability (insert); both features were lost in parallel cultures under static conditions (lower panels). B. Bone explants from an elderly (upper panels) and a 14-year-old (middle panels) healthy control were cultured in Biorector; samples were retrieved at the indicated time points and submitted to H&E staining. BM from the elderly patient is physiologically depleted of hematopoietic components, and replaced by adipose tissue. Bone trabeculae are also evident (upper panels). BM from a young donor displays a substantial cellularity, represented by hematopoietic elements and adipocytes in the context of abundant stroma (middle panels). In parallel, bone explants from the young donor, cultured in conventional static conditions, display progressive loss of both cellular and stromal components, resulting in disruption of overall architecture (lower panels). Original Magnification (OM) 200X.

We first validated the actual advantage of culturing tissue explants in Bioreactor over standard tissue culture in static conditions, setting up parallel cultures of skin and RCC explants. As shown in Figure 1, skin cultured in the RCCS™ Bioreactor for up to one week completely preserved the overall architecture, in which all strata were distinguishable. Moreover, both blood and lymphatic (D2-40 staining) vessels could be identified by morphological and immuno-histochemical analyses and preserved a patent lumen, surrounded by an intact, continuous lining of endothelial cells (EC) (Fig. 1 A, insert). Conversely, parallel cultures in static conditions exhibited consistent disruption and diffuse loss of integrity, which prevented the unequivocal identification of single strata and also vessels (Fig. 1A). RCC samples cultured in Bioreactor for up to seven days also exhibited preserved overall histoarchitecture and cell viability, at variance with those cultured under static conditions (Fig. 1A).

### RCCS™ Bioreactor is suitable for long-term culture of BM explants

It is well known that MM develops almost exclusively inside BM, so far, however, suitable models which retain native human-BM microenvironment are not available. We therefore evaluated this culture model for its ability to maintain bone explants for long-term culture. To this aim, proximal epiphyses of rat tibia were cultured either in dynamic 3D conditions (RCCS™ Bioreactor), or in 2D static conditions. As for the skin model, dynamic conditions preserved histo-architecture and cell viability inside bone explants, whereas in static conditions, deprivation of cellular component was progressively observed. As a result, cells spontaneously outgrown from tibia epiphyses began to proliferate to form a monolayer and they completely lost their native microenvironment as well as their normal organisation and morphology (Figure S2). Suitability of RCCS™ Bioreactor also for 3-D culture of MM explants, was then assessed by 3D culturing human bone biopsies from healthy donors. We analyzed samples from both elderly donors, age-matched with MM patients, and from a 14-year-old donor. Tissue explants (2–3 mm^3^ in maximal volume) were kept in 3-D dynamic culture for up to two weeks; at intervals of 3–4 days, specimens were retrieved and submitted to histopathological examination. As shown in Figure 1B, H&E staining identified intact BM architecture, consistent with age-related features, which was maintained throughout the culture period. In particular, normal bone explants, obtained from elderly subjects, displayed the classical, physiological, age-related prevalence of adipose tissue (Figure 1B, upper panel). Conversely, in BM explants from the young donor, nucleated, heterogeneous cellular elements were diffusely distributed in the context of abundant stroma (Figure 1B, middle panel). These morphological features were maintained throughout culture for up to 14 days (Figure S3A).

### 3-D dynamic culture in the RCCS™ Bioreactor of MM tissues allows the maintenance and identification of individual compartments

To validate the advantage of culture in the RCCS™ Bioreactor also for BM tissue, we set paralleled cultures of BM explants from a young donor in a conventional static condition. To this aim, we plated tissue explants of similar size in culture dishes in TCM under the same culture conditions adopted for culture in Bioreactor. When compared to matched samples from 3-D cultures carried out for up to two weeks (Figure 1B and Figure S3A), explants maintained in traditional cultures displayed progressive and striking disappearance of hematopoietic precursors and adipocytes, together with disruption of overall architecture (Figure 1B, lower panels and Figure S3B). Taken together, these data indicate that RCCS™ Bioreactor is suitable for culturing tissue explants from BM, and offers a consistent advantage over presently available, conventional tissue culture systems.

**Figure 2:**
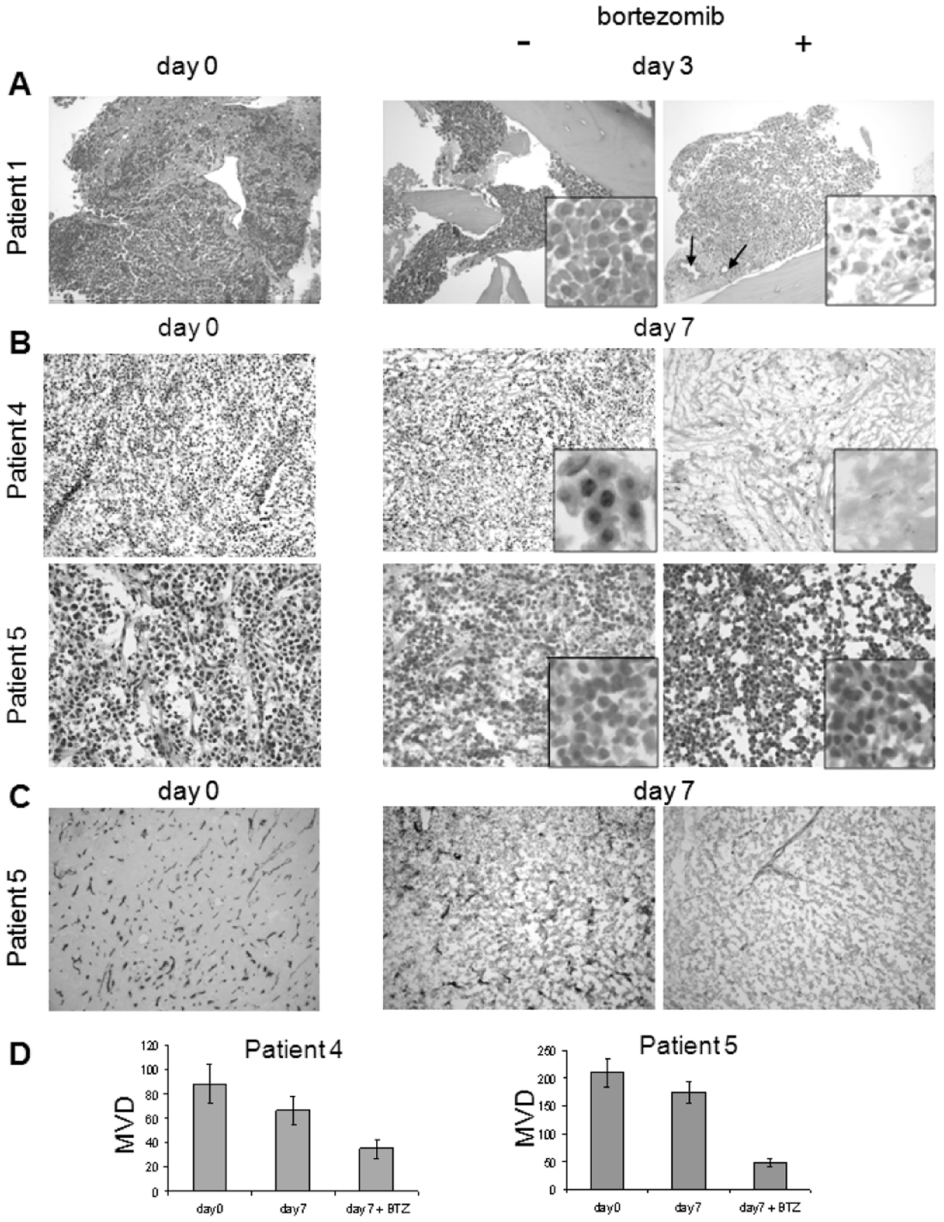
Culture in Bioreactor allows the identification of single cell components inside MM microenvironment and of their individual response to Bortezomib. MM samples from a vertebral lesion (Patient 1) and from extramedullary localizations of the Bortezomib-sensitive Patient 4 and of the Bortezomib-resistant Patient 5 were cultured in the RCCS™ Bioreactor for up to seven days in the presence (+)/absence(−) of Bortezomib 50 nM. H&E staining (Panel A and B) demonstrates the persistence of MM cells in untreated samples and the differential sensitivity to Bortezomib treatment. OM 200× for Patient 1, 100× for Patient 4 and 400 for Patient 5. Inserts are zoom of the corresponding pictures. Immunohistochemistry with anti-CD34 mAb Panel C) allows to identify vasculature and its vulnerability to Bortezomib (OM 200×). In Panel D, MVD was quantified at day 0 and after 7 days in the presence and absence of Bortezomib upon staining with CD34 mAb.

To validate this culture model for the assessment of MM microenvironment morphology as well as for the impact of drugs on its components, we utilized tissue explants from MM patients, including three bone lesions (from Patients 1, 2 and 3) and two extra-medullary lesions (from Patient 4 and Patient 5) (Table 1). Bone samples were obtained at vertebroplasty when patients were still untreated (Table 1). Histological analyses performed on BM explants cultured for three days in Bioreactor allowed the identification of single components of MM microenvironment, including MM cells, bone lamellae and vessels (arteriolae, arrows), within an overall well preserved architecture (Figure 2A). MM cells were still evident in MM explants cultured in Bioreactor for 7 (Figure 2B) and 14 (Figure S3C) days.

### 3-D culture in Bioreactor of MM explants allows the assessment of their sensitivity to Bortezomib

Our culture system was further exploited to evaluate the effects of Bortezomib treatment on MM tissue explants. To this purpose, MM specimens from vertebroplasty were cultured in Bioreactor in the presence of Bortezomib for three days, in accordance with the kinetics of anti-myeloma activity exerted by the drug *in vitro* [31]. Indeed, Bortezomib severely affected PC viability, as assessed by morphological criteria, including cell size reduction, acidophilic cytoplasmic stain, nuclear pyknosis, karyolysis and karyorrhexis (Figure 2A, inserts). Accordingly, MM cells isolated from the BM of Patients 1, 2 and 3 and cultured under static conditions appeared to be sensitive to Bortezomib treatment (50 nM) as indicated by the percentage of Annexin V+/PI+cells at FACS analysis (71%; 64% and 52%, respectively at 24 hrs, and almost 100% at three days in all cases).

To further substantiate the reliability of our culture system in discriminating the impact of drugs on MM cells and on different microenvironmental players as well, we considered samples obtained from two patients with divergent clinical history and response to therapy, *i.e.*, the Bortezomib-sensitive Patient 4 and the Bortezomib-resistant Patient 5 (Table 1). In particular, in Patient 4, a newly diagnosed MM, tissue was obtained from a skull lesion; a part of it was kept in 3-D culture, while another portion was used to isolate single PC suspension. Isolated PC resulted sensitive to Bortezomib *in vitro*, as indicated by PI incorporation at FACS analysis and by nuclear condensation and intense cytoplasmic vacuolization at TEM (Figure S4). On the other hand, patient 5 had failed several lines of therapy and was under treatment with Bortezomib when he developed subcutaneous localizations, which were excised and submitted to 3-D culture in Bioreactor. Histological analyses performed on 3-D culture samples from the two patients allowed to identify PC (Figure 2B) and to assess their differential response to Bortezomib. In Patient 4, following a 7 days treatment with Bortezomib, PC disappeared and were replaced by stroma, in agreement with the findings observed when isolated PC were exposed to Bortezomib *in vitro*. Conversely, Bortezomib treatment did not affect MM PC in tissue explants retrieved from Patient 5, a feature that corroborated the inefficacy observed *in vivo* (Figure 2B). MM-associated microvasculature was highlighted by immuno-histochemical analysis with anti-CD34 mAb (Figure 2C). Notably, the impact of Bortezomib on microvasculature inside MM microenvironment was confirmed by decreased MVD in Bortezomib-treated MM tissue explants from both Patient 4 and Patient 5 (Figure 2D), irrespective of differential cytotoxicity on MM PC.

### MM samples cultured in the RCCS™ Bioreactor express metabolic, specialized functions that may be affected by Bortezomib treatment

**Figure 3:**
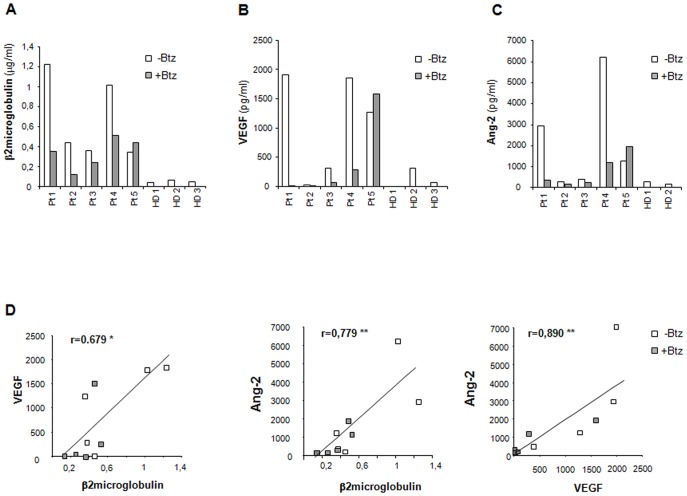
MM samples express specialized functions in culture. Supernatants from Patients' explants, cultured for 3 days in the presence (grey columns) or absence (white columns) of Bortezomib (Btz, 50 nM), were retrieved and analyzed for β2 microglobulin (A), VEGF (B) and Ang-2 (C) concentrations. In A, B and C, β2 microglobulin, VEGF and Ang-2 concentrations in three days culture supernatants from five MM samples were compared to those from three healthy donors (HD). In D, correlation between β2 microglobulin, and VEGF and Ang-2 concentrations in Patients' supernatants was assessed using the Spearman's rho coefficient (*r*). (* p<0,05; **p<0.01.)

We then assessed whether also specialized functions expressed by MM cells and their microenvironment were maintained in 3-D culture in the RCCS™ bioreactor. To this aim, we first looked at β2 microglobulin concentration in culture supernatants from MM explants retrieved at different time intervals. Indeed, β2 microglobulin serum levels significantly correlate with PC proliferation index [32] and are commonly used for staging of MM. As shown in Figure 3A, β2 microglobulin was detectable in supernatants obtained after three days of culture; moreover, β2 microglobulin levels decreased in the presence of Bortezomib in all samples, but in patient 5, in accordance with the drug sensitivity of MM cells shown by morphological examination. β2 microglobulin levels were instead negligible in supernatants from healthy bone explants of comparable size derived from 3 controls (HD) and cultured in the same experimental conditions (Figure 3A).

Overproduction by MM cells of pro-angiogenic cytokines, including VEGF and Ang-2, is responsible for MM-induced angiogenic switch and in turn for MM progression [15]–[17], [33]. Indeed, VEGF and Ang-2 could be detected in supernatants after three days of culture; moreover, their secretion was inhibited upon Bortezomib treatment with the exception of patient 5, closely paralleling the results obtained with β2 microglobulin release. Again, supernatants from controls contained very low levels of both VEGF and Ang-2 (Figure 3B–C). Notably, VEGF and Ang-2 expression positively and significantly correlated with that of β2 microglobulin (*r* = 0.679; p<0.05; *r* = 0.779; p<0.01, respectively) and also correlated with each other (*r* = 0.0890; p<0.01) (Figure 3D).

**Figure 4:**
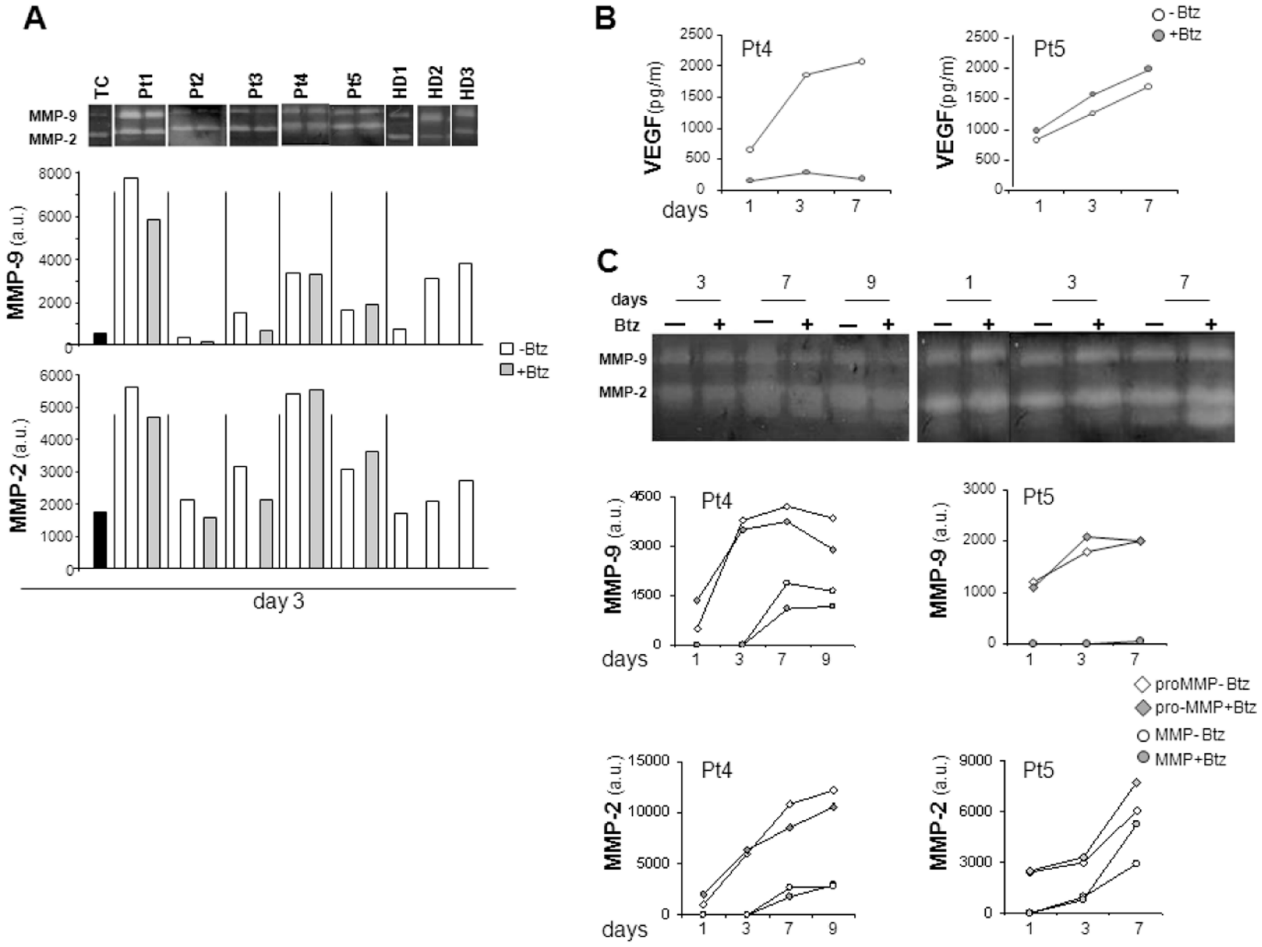
Kinetics of MMP-9 and MMP-2 activities and of VEGF secretion in culture supernatants. A: Supernatants from biopsies of 5 MM Patients and 3 healthy donors (HD), cultured for 3 days in the presence or absence of Bortezomib (Btz, 50 nM), were assessed for MMP-2 and MMP-9 activities. Zymography of both MMPs is shown (upper panels) and quantitated by densitometry (lower panels). White columns represent samples obtained in the absence of Bortezomib while grey columns in the presence of the drug. TC, black columns, represent negative controls. Kinetics of VEGF (B), pro-MMP and MMP activities (C) were determined in supernatants obtained from Patient 4's and Patient 5's samples. MM explants were kept in culture in bioreactor in the presence (grey) or absence (white) of Bortezomib (Btz, 50 nM); supernatants were retrieved at different time intervals and submitted to ELISA for VEGF determination (B) and to zymographic analysis (C) to assess MMP-9 and MMP-2 enzymatic activities. a.u. = arbitrary units.

MM is characterized by accelerated production of the proteolytic enzymes MMP, which are critically involved in tumor growth, angiogenesis, homing and the development of osteolytic lesions, all typical events encountered in MM progression [34], [35]. Zymography performed on day three supernatants from MM tissue explants showed MMP-2 and, to a lesser degree, MMP-9 activity. Both MMP-2 and MMP-9 activity varied among patients, as did the response to Bortezomib treatment (Figure 4A). Supernatants from controls also contained measurable MMP activity, which was substantially lower than that from patient's samples in the case of MMP-2, while did not differ from that of most patients in the case of MMP-9.

### Long-term maintenance of MM specialized functions in the RCCS™ Bioreactor

To investigate whether culture in Bioreactor sustains proper differentiated functions over time, we took advantage of samples derived from the prototypical Patients 4 and 5. Supernatants obtained at different time points showed a progressive increase in VEGF concentration (Figure 4B). Notably, VEGF concentration abated in the presence of Bortezomib in the case of Patient 4, while it was unaffected in the case of Patient 5, once again paralleling the sensitivity to the drug of MM cells (Figure 4B). MMP-2, and to a lesser degree MMP-9 activity, also increased in supernatants from both patients for up to day 7, when a partial conversion of the proMMPs into their activated form can be observed. In Patient 4, Bortezomib treatment mildly affected both pro and active MMPs, while in Patient 5 no effect could be determined (Figure 4C). MMP-9 and -2 activities were also detectable in supernatants from HD samples; however, their levels did not increase over time and the active form could not be appreciated (Fig. S3 D).

### Effect of Bortezomib on β2 microglobulin levels in patients' sera and supernatants from cultures in Bioreactor

**Figure 5:**
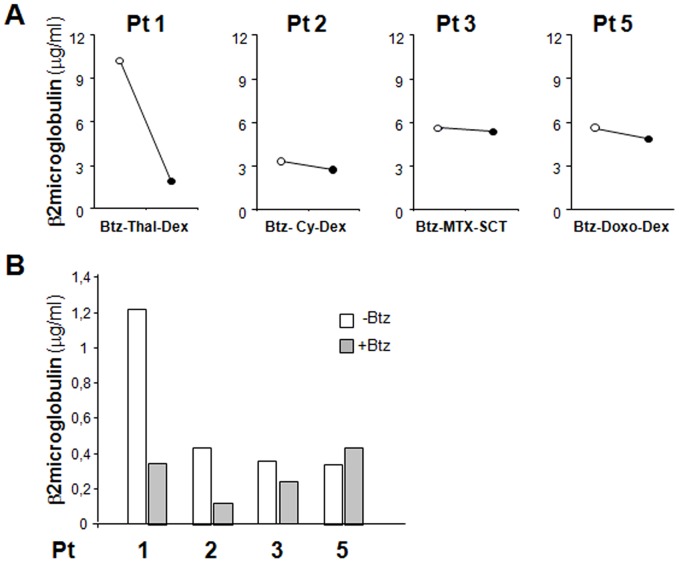
β2 microglobulin levels in patients'sera and in supernatants from cultures in the RCCS™ Bioreactor. A: β2 microglobulin concentrations were determined in patients'sera before (white circles) and after (black circles) Bortezomib-based therapies (×axis). B: β2 microglobulin levels in 3 days supernatants from MM explants cultured in bioreactor in the absence (white) or presence (grey) of Bortezomib. Abbreviations: Bortezomib, Btz; Thal, thalidomide; Dex, Dexamethasone; Cy, Cyclophosphamide; Mel, Melphalan; HSCT, Hematopoietic Stem Cell Transplantation; Doxo, Doxorubicin.

Finally, to investigate the suitability of culture in Bioreactor to predict sensitivity to drugs, we compared β2 microglobulin levels in patients' sera before and after Bortezomib-based therapies with those observed in supernatants from cultures in presence/absence of Bortezomib. In particular, we analyzed Patients 1, 2, 3 and 5; Patient 4 was not included, since she did not receive Bortezomib treatment (see Materials and Methods section). Figure 5 demonstrates an overall concordance between the effects exerted by Bortzomib *in vivo* and *ex vivo*.

## Discussion

We herein demonstrate that some important biological aspects of MM, and response to drugs of individual cellular components inside MM microenvironment, can be reliably evaluated with a 3-D culture system in RCCS™ Bioreactor. Among all available 3-D culture methods, culture of normal and pathological tissues represents the most relevant, since it enables the *ex vivo* study of cellular processes, in a multicellular context in which the original tissue architecture and microenvironment are maintained [23], [36]. Currently available conventional static and dynamic culture systems (shacking or rolling platforms) fail to provide optimal gas/nutritional support (and waste removal) thus resulting in reduced viability of tissue explants and impaired cell morphology and function [37]–[38]. Accordingly, the comparison of normal (skin biopsies and BM explants) as well as cancer tissues cultured in static *vs* dynamic conditions provided by the RCCS™ Bioreactor clearly demonstrates the advantage of the latter, in terms of cellularity and histo-architecture.

The dynamic RCCS™ bioreactor-based tissue culture method, taking advantage on the unique milieu generated by simulated microgravity conditions [39], [40], characterized by low shear and turbulence, by optimal O_2_ and nutrients delivery and waste removal [41], allows culture of MM explants, preserving the proper topographic and functional interactions between myeloma cells and the hosting microenvironment. In such conditions, tissue culture could be maintained for up to two weeks, thus substantially extending the duration (few days) achieved with currently available methods [38], [39]. The model could be further implemented to recapitulate more closely physical features of the BM, and in particular hypoxia [42], whose impact on MM cell behaviour and response to drugs deserves to be investigated.

Notably, histological analyses performed on serially retrieved MM explants allow the monitoring of the response to anti-myeloma drugs. This was particularly evident when samples from two prototypical Patients, were examined. Our 3-D culture system also offers the unique opportunity to assess the response to drugs of native MM associated vessels, whose structure and quantification over time can be only partially evaluated in conventional culture systems. Indeed, the 3-D model previously described by Kirshner et al. [14], based on the *in vitro* reconstruction of MM microenvironment, fails to recapitulate the original structure of the tissue vasculature. More recently, an additional 3-D model of human MM microenvironment has been developed, based upon the implantation into a SCID mouse of polymeric scaffolds coated by human BM stromal cells (BMSC) and then repopulated *in vivo* with human MM cells [43]. This model represents a further advance, especially for preclinical evaluation of anti-MM agents in a proper microenvironmental context. Also in this case, however, vessels are neo-formed inside engrafting BMSC.

While several anti-myeloma agents, including Bortezomib, are endowed with anti-angiogenic properties, the actual effects on MM–associated vessels cannot be easily estimated [22]. Specifically, neither imaging techniques nor reliable bio-markers of ongoing angiogenesis, which are being applied especially to solid tumors, have been validated so far for MM [22]. In the present RCCS™-based model, the anti-angiogenic effect of Bortezomib inside MM microenvironment could be appreciated and measured by means of MVD quantification. Notably, we have previously shown that the cytotoxicity exerted by Bortezomib *in vitro* was directed against proliferating/activated EC, reminiscent of MM-associated EC [44], but not against quiescent EC [21].

Specialized functions of MM cells and microenvironment could be assessed in supernatants from MM explants cultured in the RCCS™ Bioreactor. β2 microglobulin, VEGF and Ang-2, which are released by MM cells, positively and significantly correlated with each other, as already demonstrated in MM sera [45]. Moreover, all factors were reduced upon culture with Bortezomib, concomitantly with PC death. Members of the MMPs family are also expressed in MM microenvironment and are implicated in tumor growth and dissemination, angiogenesis and development of osteolytic lesions. In particular, MMP-9 and possibly MMP-2 are synthesized by MM cells, which also contribute to the activation of the latent forms [27], [46]. Accordingly, MMP-9 and -2 were expressed and increased over-time in supernatants; notably, the active forms were detectable during culture, indicating the existence of efficient cell-to-cell interactions. MMP-9 and -2 activities were also found in supernatants from HD, as reported in individuals with hip fractures [47] where they are implicated in fracture healing; however, their levels did not increase over time, neither the active forms could be distinguished (Fig. S3 D). Variations in MMP levels in response to Bortezomib treatment overall mirrored that of MM cells; however, MMPs appeared only slightly affected, supporting the notion that their production and activities result from additional cellular components inside MM microenvironment, including EC, BMSC, and osteoclasts [27], [34], [35]. Finally, determination of β2 microglobulin levels in patients'sera and in supernatants from Bioreactor disclose an overall concordance in the response to Bortezomib treatment; these data, albeit preliminary given the limited number of patients analyzed and the heterogeneity of Bortezomib-based therapies applied, suggest the possibility to exploit the model on a larger series to predict sensitivity to drugs in individual patients.

In conclusion, our findings indicate that the RCCS™ Bioreactor permits culture of MM explants allowing assessment of metabolic activity and drugs sensitivity of MM cells and their microenvironment, particularly vessels. This model complements currently available models for the study of MM cells-BM interaction [48]; moreover, it can be further exploited for screening of new anti-myeloma drugs [49] and for a pre-clinical approach to patient-targeted therapy of MM.

## Supporting Information

Figure S1 **Major characteristics and advantages of dynamic culture in Bioreactor in comparison to the static one.**
(TIF)

Figure S2 **Dynamic culture in Bioreactor of rat tibial explants preserves histo-architecture.** Tibial proximal epiphyses from young rats were submitted to dynamic, 3-D culture in RCCS Bioreactor for up to two weeks, retrieved at weekly intervals and stained with H&E. Evidence of hematopoietic elements and adipose tissue inside a well preserved BM architecture is assessable throughout the culture period, while in static conditions the majority of cells leave BM already at 7 days and progressively formed a classical 2-D monolayer culture. OM: 200× for 3-D culture, 400× for 2-D culture.
(TIF)

Figure S3 **Long-term culture of normal BM and MM samples in Bioreactor.** BM samples from a young donor were splitted in parallel dynamic (A) and static (B) cultures and maintained for 14 days. In C, MM sample from patient 5 was kept in culture for up to 2 weeks in Bioreactor. OM: 200×; insert is a zoom of the corresponding picture. In D, kinetics of MMP-9 and -2 activities in supernatants from healthy donors (HD); zymographic analyses, upper panels; densitometry, lower panels.
(TIF)

Figure S4 **Isolated MM cells from Patient 4 are sensitive to Bortezomib** ***in vitro*** **.** **A**: MM cells isolated from a skull lesion were treated for 24 hrs with Bortezomib (50 nM). CD138+ PC death was determined by FACS analysis by Propidium Iodide (PI) staining. **B**: TEM on MM cells treated with Bortezomib (50 nM) for 24 hrs shows nuclear condensation and massive cytoplasmic vacuolization, at variance with control (N.T.), that displays intact nuclei and cytoplasmic organelles.
(TIF)
