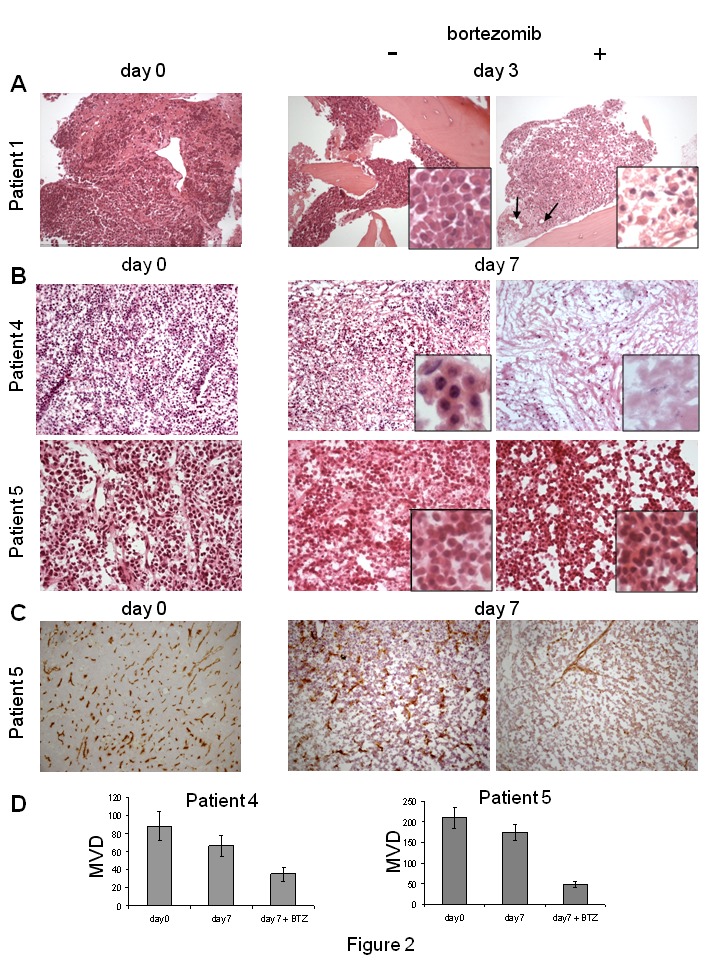# Correction: *Ex-Vivo* Dynamic 3-D Culture of Human Tissues in the RCCS™ Bioreactor Allows the Study of Multiple Myeloma Biology and Response to Therapy

**DOI:** 10.1371/annotation/d7d8e0a7-aa3d-4620-98e5-c5a7bbf31dc8

**Published:** 2013-10-25

**Authors:** Marina Ferrarini, Nathalie Steimberg, Maurilio Ponzoni, Daniela Belloni, Angiola Berenzi, Stefania Girlanda, Federico Caligaris-Cappio, Giovanna Mazzoleni, Elisabetta Ferrero

Correct version of Figures 1 and 2 are available below.

Figure 1: 

**Figure:**
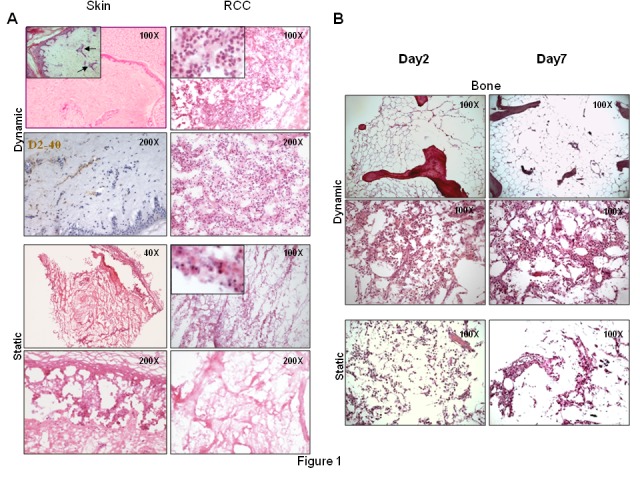


Figure 2: 

**Figure:**