# Supplementary figures and images for: Ex-Vivo Dynamic 3-D Culture of Human Tissues in the RCCS™ Bioreactor Allows the Study of Multiple Myeloma Biology and Response to Therapy
Source: PLoS One. 2013 Aug 26;8(8):e71613. doi: 10.1371/journal.pone.0071613 (PMC3753321; doi:10.1371/journal.pone.0071613)

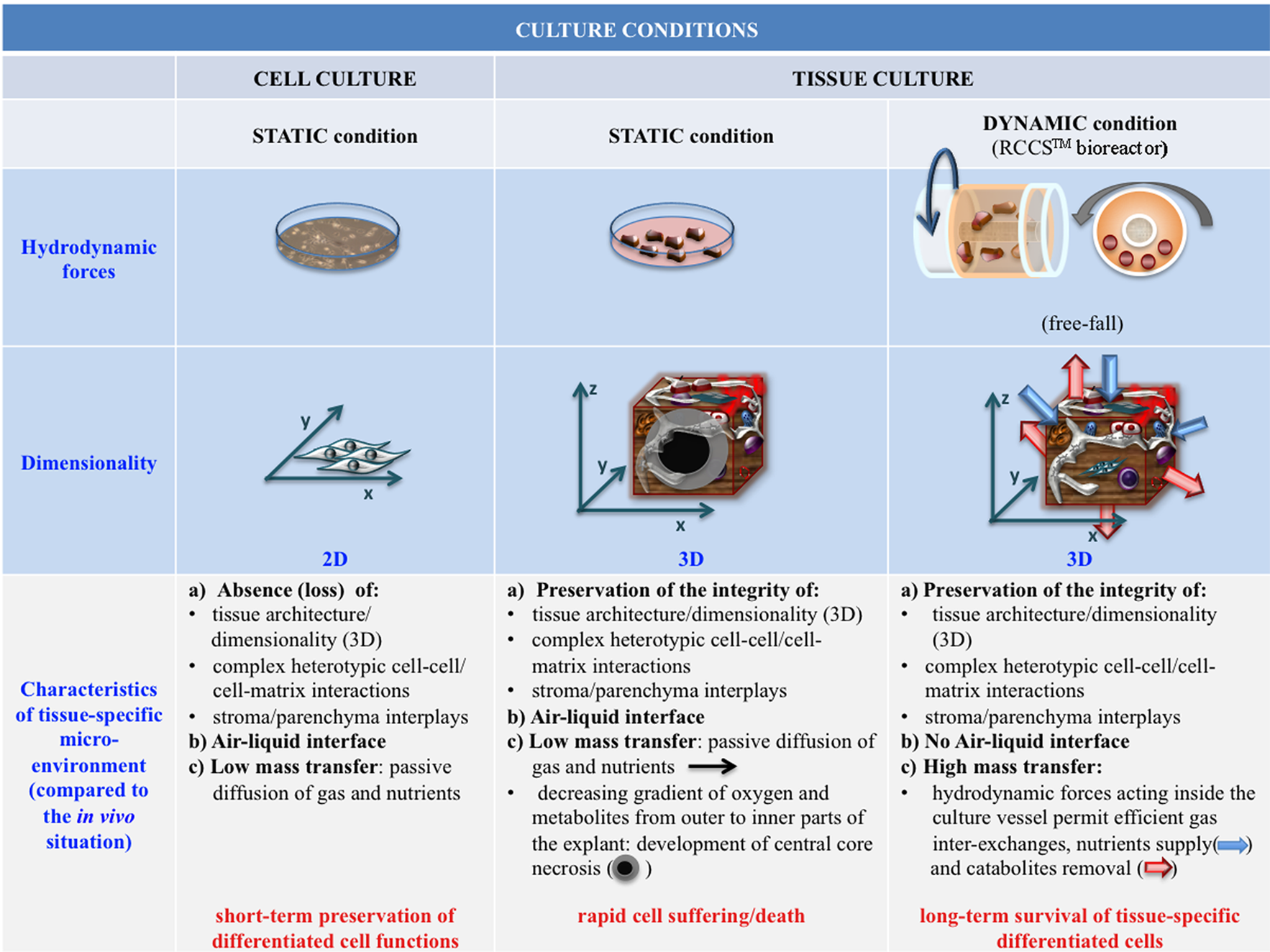

Supplement: Figure S1 — Major characteristics and advantages of dynamic culture in Bioreactor in comparison to the static one. (TIF) [file pone.0071613.s001.tif]

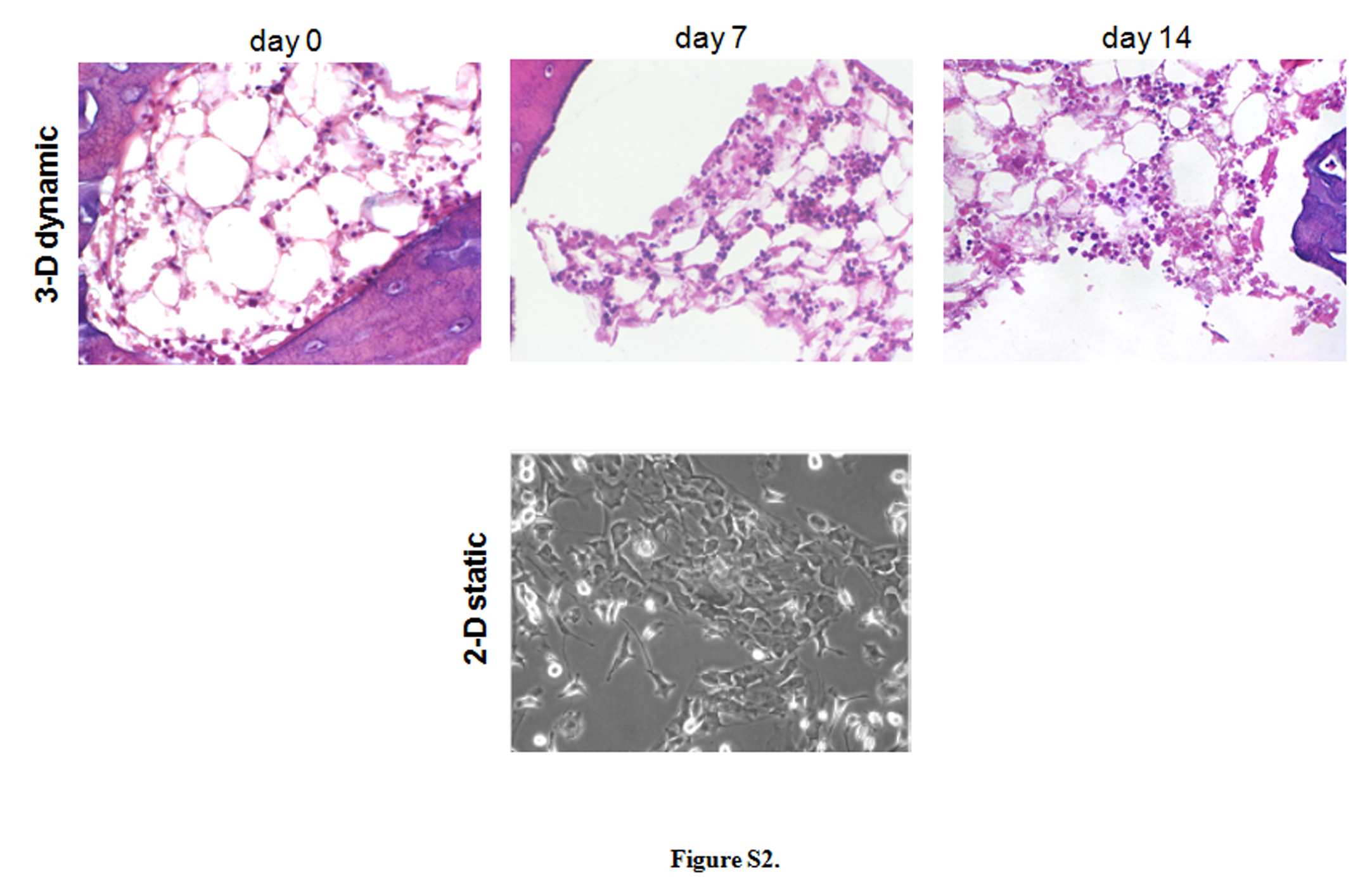

Supplement: Figure S2 — Dynamic culture in Bioreactor of rat tibial explants preserves histo-architecture. Tibial proximal epiphyses from young rats were submitted to dynamic, 3-D culture in RCCS Bioreactor for up to two weeks, retrieved at weekly intervals and stained with H&E. Evidence of hematopoietic elements and adipose tissue inside a well preserved BM architecture is assessable throughout the culture period, while in static conditions the majority of cells leave BM already at 7 days and progressively formed a classical 2-D monolayer culture. OM: 200× for 3-D culture, 400× for 2-D culture. (TIF) [file pone.0071613.s002.tif]

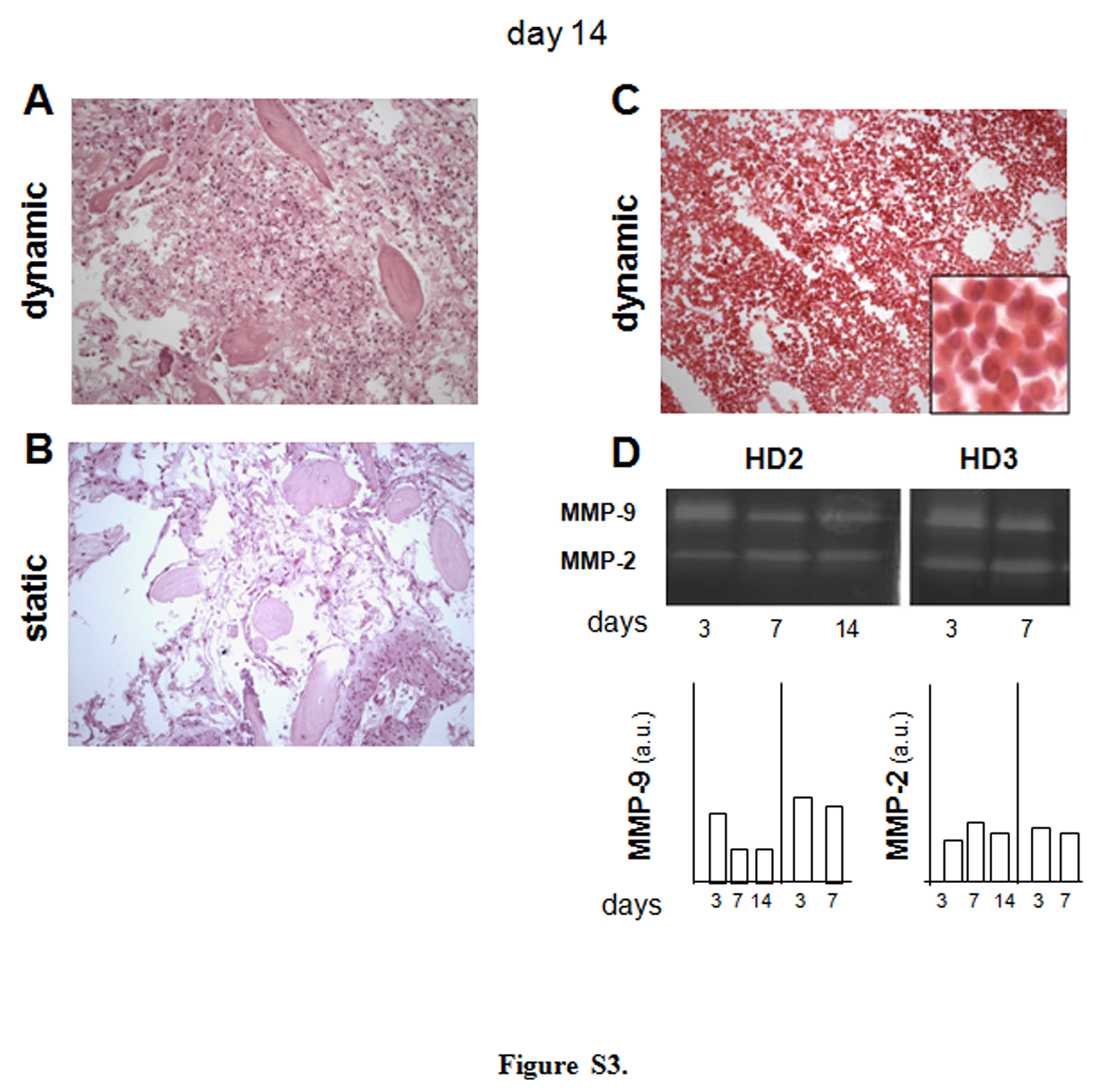

Supplement: Figure S3 — Long-term culture of normal BM and MM samples in Bioreactor. BM samples from a young donor were splitted in parallel dynamic (A) and static (B) cultures and maintained for 14 days. In C, MM sample from patient 5 was kept in culture for up to 2 weeks in Bioreactor. OM: 200×; insert is a zoom of the corresponding picture. In D, kinetics of MMP-9 and -2 activities in supernatants from healthy donors (HD); zymographic analyses, upper panels; densitometry, lower panels. (TIF) [file pone.0071613.s003.tif]

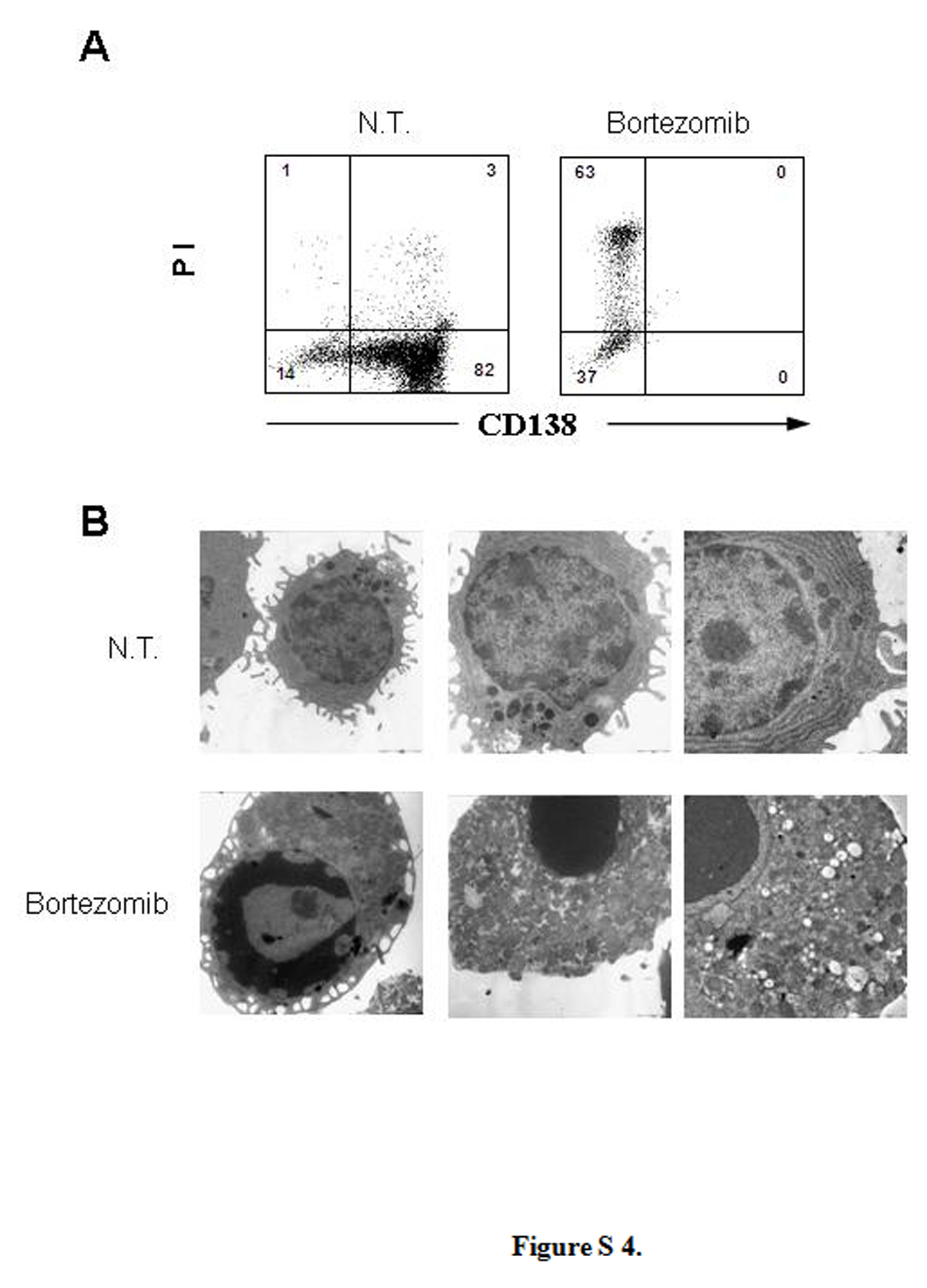

Supplement: Figure S4 — Isolated MM cells from Patient 4 are sensitive to Bortezomib in vitro . A: MM cells isolated from a skull lesion were treated for 24 hrs with Bortezomib (50 nM). CD138+ PC death was determined by FACS analysis by Propidium Iodide (PI) staining. B: TEM on MM cells treated with Bortezomib (50 nM) for 24 hrs shows nuclear condensation and massive cytoplasmic vacuolization, at variance with control (N.T.), that displays intact nuclei and cytoplasmic organelles. (TIF) [file pone.0071613.s004.tif]
